# Vaginal drug delivery for the localised treatment of cervical cancer

**DOI:** 10.1007/s13346-017-0395-2

**Published:** 2017-06-08

**Authors:** Ian Major, Christopher McConville

**Affiliations:** 1 Materials Research Institute, Athlone Institute of Technology, Athlone, Ireland; 2 School of Pharmacy, Institute of Clinical Sciences, College of Medical and Dental Sciences, University of Birmingham, Edgbaston, Birmingham, UK

**Keywords:** Cervical cancer, Fertility sparing surgery, Neoadjuvant chemotherapy, Vaginal drug delivery, Localised drug delivery

## Abstract

Cervical cancer is usually treated by surgery, with the more advanced cancers requiring adjuvant chemotherapy or radiotherapy. The location of the cervix makes it easily accessible through the vagina for the localised delivery of chemotherapeutic drugs. Localised delivery has the advantage of direct delivery to the site of action resulting in a lower dose having to be required and a reduction in systemic side effects. This approach would be advantageous for fertility sparing surgery, whereby localised delivery could be used to reduce tumour size allowing for a much smaller tumour to be removed, reducing the risk of preterm birth. Furthermore, localised delivery could be used after surgery to reduce the risk of recurrence, which is significantly higher in fertility sparing surgery compared to standard surgery. In this paper, we discuss the number of vaginal dosage forms that have investigated for this purpose, including tablets, rings, bioadhesive and cervical caps. APIs under investigation have ranged from well-established chemotherapeutic drugs to more experimental compounds.

## Introduction

Cervical cancer is the third most prevalent cancer in women globally, with 528,000 cases a year [1]. A total of 85% of cases arise in developing countries due to a lack of cervical cancer prevention and control programs. Where women have access to resources capable of detecting and treating precancerous lesions, the number of cases is reduced by approximately 80% [2]. The sexual transmission of the human papillomavirus (HPV) is the main cause of cervical cancer. Other risk factors, such as multiple sex partners, smoking, sexual activity at a young age, multiple pregnancies, oral contraceptives and other sexually transmitted infections, can also contribute to cervical cancer [3, 4].

## Epidemiology

In 2012, there was an estimated 266,000 deaths from cervical cancer worldwide [1]. There is a remarkable disparity in the effects of disease between different parts of the globe [5]. The most impacted world area is Eastern Africa where the age-standardised mortality rate is 27.6 per 100,000. The least impacted world area is Western Asia where the age-standardised mortality rate is 1.9 per 100,000. Since cervical cancer is a preventable disease and screening programs are commonplace, the rates of mortality and incidence have dropped considerably in the developed world since the mid-seventies. For example, in Denmark, the age-standardised incidence rate has fallen from 70.9 per 100,000 to 19.2 per 100,000 over four decades [6]. The mortality rate for the disease in Denmark stands at 2.6 per 100,000 [7]. Denmark began a national roll-out of cervical screening in 1967 following on from a small population-based study in a single municipality begun in 1962 [8]. The total roll-out completed in 1996. A recent study determined that the Danish screening program prevented 51.7% of the projected cumulative incidence cases over the period of 1961 to 2010 [6], which would be in the region of 27,506 cases.

African nations are faring much worse in cervical cancer prevention as African countries constitute 16 of the top 20 countries with the highest incidence rates of the disease [1]. Malawi has the highest incidence rate of 75.9 per 100,000 in the world, with a mortality rate of 49.8 per 100,000. The country has had an active prevention program since the late eighties [9], and almost all the country’s health districts provide screening services using the WHO-approved visual inspection with acetic acid (VIA) [10]. However, very few of the 4.50 million at-risk women were screened with participation being 3.7% urban women and 2.5% rural women. One possibility for low participation in the national screening program may be due to roll-out having been restricted to district hospitals with only limited extension of the services to local health centres. An in-depth study by the University of Malawi cited a lack of adequately trained healthcare staff and that the existing staff were not sufficiently distributed or well-supervised [11]. The study described a lack of basic screening and treatment equipment and medical consumables in the health facilities running providing services.

However, the Malawi experience of a national screening program cannot easily be overlaid onto other African and low-resource countries since there are infrastructural limitations and cultural barriers unique to each country and region. Alliance for Cervical Cancer Prevention (ACCP) has made a number of recommendations for improving the efficiency and quality of cervical cancer screening and treatment in low-resource settings with the aim of decreasing high mortality rates [12]. The AACP study included the analysis of data from a wide variety of world areas. The AACP makes a number of recommendations, including a move away from cytology-based screening towards HPV-DNA testing that is more cost-effective and laboratory-independent. The study also recommends a single visit approach in which the patient is tested and treated on the same occasion, thus reducing the high loss to follow-up rates currently experienced. Treatment would come in the form of cryotherapy for precancerous lesions. Limited access to the two expensive HPV vaccines (Gardasil® and Cervarix®) may also be a factor in the higher mortality rates in low-resource settings [5], but there is of yet no data to affirm primary prevention of HPV-related cancers for the large populations that have rolled-out a vaccine program.

## Staging

Cervical cancer, as with most cancers, has been classified into stages so as to better manage patients and standardise treatment [13]. In carcinoma in situ (stage 0), abnormal cells are found in the innermost lining of the cervix and may become cancerous and spread into nearby normal tissue. Stage I cancers are only located at the cervix and depending on the amount of cancer found can be divided into stages IA and IB, which can be sub-divided into stages IA1, IA2, IB1 and IB2 depending on the size of the tumour. Stage II cancers will have spread beyond the cervix, but not as far as the pelvic wall or the lower third of the vagina and are divided into IIA and IIB, based on how far the cancer has spread. In stage III, the cancer has spread to the lower third of the vagina and/or pelvic wall and may also have started to cause kidney problems. Like stage II, stage III is also divided into stages IIIA and IIIB, based on how far the cancer has spread. By stage IV, the cancer has spread to the bladder, rectum or other parts of the body. Stage IV can also be divided into IVA and IVB, depending on where the cancer is found. In stage IVA, the cancer has spread to nearby organs such as the bladder and rectum, while in stage IVB, the cancer may be found in other parts of the body, such as the liver, lungs, bones or distant lymph nodes. There are currently two approaches used to prevent cervical cancer from developing to an advanced stage (stage IIB): (1) the distribution of HPV vaccines that are mainly directed against HPV types 16 and 18 [14] and (2) screening methods, such as Papanicolaou test (Pap smear), which involves the collection of exfoliated cells from the cervix, which are then examined for cellular abnormalities enabling the identification of CIN before they begin to develop into cervical cancer [15].

## Treatment options

Following the diagnosis of cervical cancer, there are many different treatment options available all of which depend on the stage of the cancer and the health of the patient. Pre-invasive (stage 0) and early stage cancers (IA to IIA1) are treated using surgery such as loop electrosurgical excision procedure (LEEP), large loop excision of the transformation zone (LLETZ) or cone-shaped excision, which involve the removal of a small amount of cervical stroma [16], or a hysterectomy (removal of the whole uterus including part of the vagina) and removal of the lymph nodes or using radiation therapy. Patients treated using surgery may also receive radiation therapy in order to reduce the risk of relapse. Larger early stage cancers (IB2 and IIA2) are treated with either radiation therapy or chemotherapy or a hysterectomy followed by radiation therapy, or chemotherapy followed by a hysterectomy. Advanced stage cancers (IIB to IVB) are treated using radiation therapy and chemotherapy [17]. Cisplatin is deemed the single most active agent against cervical cancer [13]. The options for chemotherapy are cisplatin once a week during radiation or cisplatin with 5-fluorouracil once every 4 weeks during radiation.

### Radical hysterectomy

Hysterectomy is an all-encompassing term that is used to describe surgical procedures of various degrees of radicality. Treatment of cervical cancer by surgical intervention has a long history dating well back into the 1800s, and significant refinement in approach was observed throughout the first half of the last century to improve post-operative mortality rates and quality of life [18]. Yet, the first attempt to have a standardised form of surgical treatment employing classification has only been a fairly recent development [19]. Surgical classification aims to provide a basis for surgery taking into account a number of prognostic considerations aiming to maximise the removal of cancerous tissue but also to limit damage to important cervicovaginal structures and function. Surgeons take consideration of tumour depth and spread, status of the surrounding lymph nodes and lymphovascular space invasion [18].

The first widely adopted classification system dates to 1974 and the work of a group of surgeons at MD Anderson in Texas [19]. This system adopted five classes of radical hysterectomies that increased in radicality up the scale. Class I involved removal of the cervical tissue only, while class V involved the removal of the cervix, pelvic lymphatics and extensive amounts of paravaginal, parametrial and periureteral tissues, plus sections of the ureter and bladder. The system lacked clear anatomical definitions and did not describe methods for nerve preservation. Nerve preservation is a key consideration area since it has been shown to provide a significant reduction in morbidities associated with radical hysterectomy, including loss of sexual, bladder and anorectal functions [20–24], and reduced vaginal blood flow [25]. However, this approach may limit the curative effect of surgery since the remaining nerves and connective tissue may be sites for local recurrence. Two recent systematic reviews were both inconclusive in this regard due to limitations of previous studies [26, 27].

Critical of the previous classification system, Querleu and Morrow devised their own in 2008 with the desire of providing a simpler, clearer approach that reduced excessive vaginal resection and comprehensively preserved more nerves [28]. This newer system adopts clear anatomical markers by adopting international definitions and creates four types of classifications: (a) minimum resection of paracervix, (b) transection of paracervix at the ureter, (c) transection of paracervix at junction with internal iliac vascular system and (d) laterally extended resection. The system considers lymph node resection separately and is based around the stable arterial anatomy for orientation as opposed to the older system’s use of spatial descriptors. Vaginal resection and ureter management are modifiable components of each type and are to be determined by the surgeon for each individual case. A follow-up consensus paper adds to the original by describing individual three-dimensional parametrial resections for a number of the classification types [29].

## Localised delivery of chemotherapeutic drugs

**Fig. 1:**
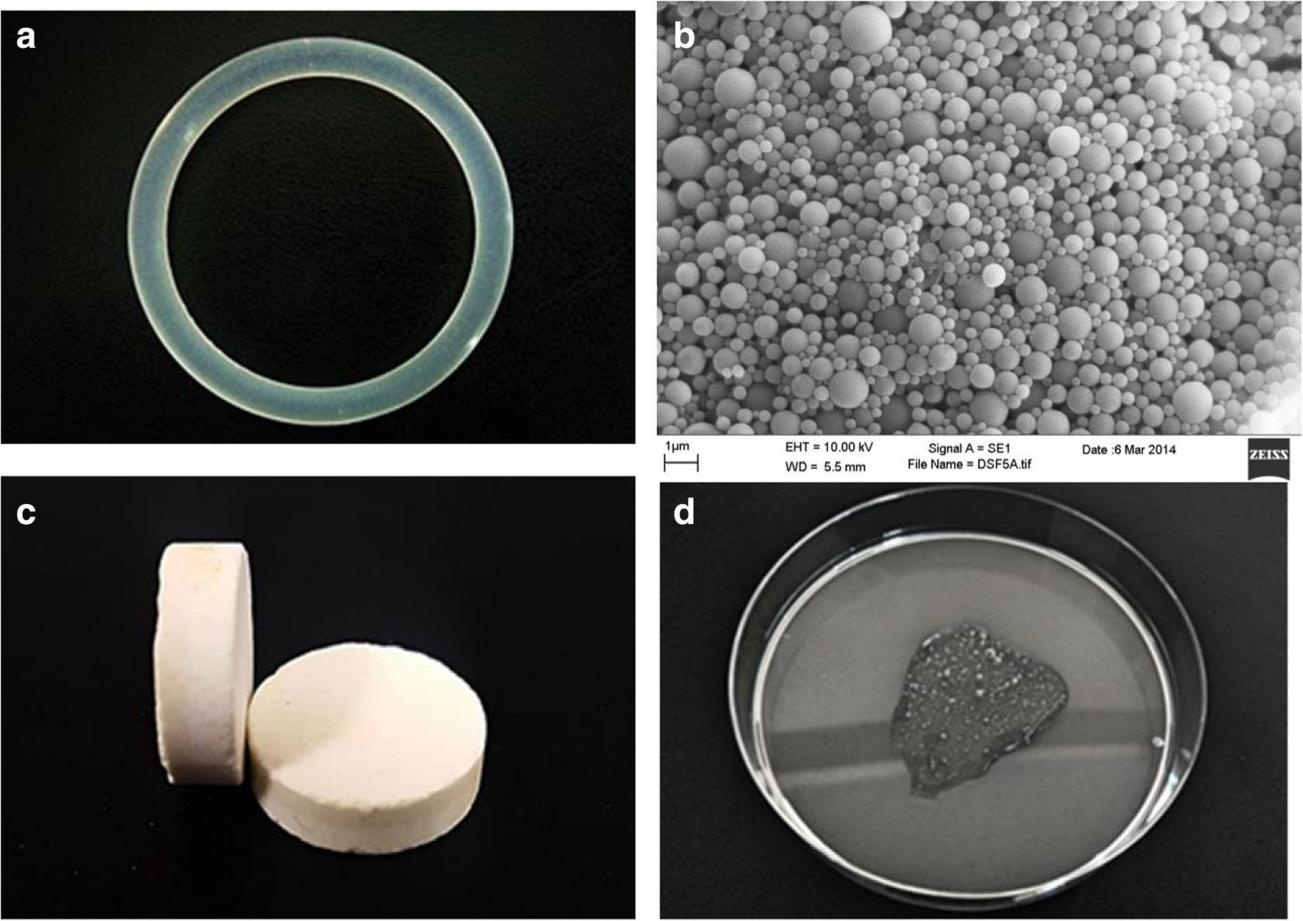
Typical vaginal dosage forms for the delivery of chemotherapeutic drugs **a** ethylene vinyl acetate copolymer vaginal ring with an outer diameter of 54 mm and a cross-sectional diameter of 4 mm. **b** Nano-encapsulated disulfiram particles (1 μm scale). **c** Bilayer vaginal tablets (13 mm diameter and 4 mm thick) containing a disulfiram loaded sustained layer and copper (II) sulphate immediate release layer. **d** Hydroxyethylcellulose based vaginal gel, typical 2 g application on Petri dish

Localised delivery of chemotherapeutic drugs offers a number of advantages over systemic administration: (1) direct delivery to the site of action, (2) a lower dose being required, (3) a reduction in systemic side effects and (4) increased drug stability as it remains in the delivery device until released [30]. The location of the cervix makes it easily accessible through the vagina and allows for the non-invasive implantation of a localised drug delivery device or formulation adjacent to the cancerous tissue either before resection, to reduce tumour size, or after resection to reduce the risk of recurrence. Furthermore, cervical cancer is an excellent choice for localised drug delivery as due to regular screening approximately 50% of cases are diagnosed when the cancer is confined to the cervix (localised; stage I), while about 35% of cases are diagnosed after the cancer has spread to the lymph nodes (regional; stage II/III). Therefore, only about 10% of cases are diagnosed when the cancer has already spread to distant regions (metastasized; stage IV). Localised drug delivery is only an option for stage 0 (precancerous), stage I, which are still confined to the cervix and early stage II cancers, which have spread beyond the cervix, but not as far as the pelvic wall or the lower third of the vagina. For late stage II through to stage IV, cervical cancer systemic delivery of chemotherapeutic drugs would be required to ensure that the drug reaches those cancer cells which have spread to other parts of the body such as the bladder, lungs or lymph nodes (Fig. 1).

### Vaginal drug delivery for the localised treatment of cervical cancer

The vagina has been used to deliver drugs for a range of clinical and research applications, including contraception, vaginal infections and HIV prevention, with many different vaginal formulations such as gels, creams, pessaries, suppositories, diaphragms, rings, films and tablets available [31–39]. Furthermore, a number of these delivery systems have been investigated for the localised delivery of chemotherapeutic drugs to the cervix [40–44]. When considering the vaginal route of administration for localised delivery, two key areas need to be considered: (1) the nature of the drug being administered and (2) the uterine first pass effect.

The nature of the drug is important when it comes to choosing the dosage form that will be used for delivery. For example, conventional vaginal rings are only capable of delivering small hydrophobic molecules due to the type of polymers which are used to manufacture vaginal rings. For a drug to be released from a vaginal ring, it must first dissolve in the polymer matrix (which is manufactured from hydrophobic polymers such as silicone and ethylene-vinyl-acetate copolymer) and subsequently diffuse through the polymer (large molecules will not diffuse through the polymer matrix). However, once the drug leaves the vaginal ring, it must dissolve in the aqueous vaginal fluid and thus must have some aqueous solubility. Therefore, it is recommended that for a drug to be formulated into a vaginal ring, it must have a molecular weight of less than 1000 Da and a partition coefficient (log P) of between approximately 2 and 4. For other dosage forms such as vaginal gels and tablets, the molecular weight of the drug is not so critical in relation to drug release, whereas the log P is critical, because the drug needs to be hydrophilic enough to dissolve in vaginal fluid, but hydrophobic enough to be absorbed into vaginal tissue and the site of action.

The uterine first pass effect is where vaginally administered drugs preferentially accumulate in the uterus through a direct transport mechanism [45, 46]. The effect has been described for a number of drugs, principally progesterone [47, 48] and other steroids [49–51]. Direct vaginal-to-uterine transport is thought to occur via the counter-current exchange of drug through vein-to-artery diffusion [52]. The phenomenon is driven by the close proximity of a dense venous network in the upper vagina to the uterine artery, in which the high concentration of drug in the vaginal blood vessels will diffuse across into the adjoining uterine artery. Blood circulation is a key mechanism of the uterine first pass effect, since plasma drug concentrations have been shown to be significantly higher in the uterine artery than in the radial artery [48]. Such direct transport has the potential to be a major issue for therapies required to act locally in the vagina as the drugs could be transported away, thus unable to achieve effective concentration levels at the site of action. Since the uterine first pass effect only occurs in the upper third of the vagina [49], the phenomenon may significantly hinder localised delivery to the cervix. Therefore, larger doses may be needed to achieve effective drug levels at the cervix. Furthermore, with the accumulation of the drug in the uterus and an increase in the direct transport of the drug from the vagina to the uterus as a result of higher concentrations in the vagina, toxicity could be a potential problem, especially with chemotherapeutic drugs.

### Topical formulations

Localised delivery does not currently form part of standard treatment of cervical cancer. However, off-label use of both 5-fluorouracil (5-FU) and imiquimod (IMQ) topical creams can form part of treatment of genital intraepithelial neoplasia at the discretion of the treating physician [53–57]. 5-FU is an antimetabolite drug that has two mechanisms of action—inhibition of the thymidylate synthase enzyme and misincorporation into DNA and RNA [58]. Since the drug’s first synthesis in the fifties, it has been widely used to treat a number of cancers, most notably colorectal. Topical formulation of the drug is used to treat skin cancer and HPV-related warts, lesions and neoplasia [55, 59–61]. Rahangdale et al. conducted a clinical study to evaluate the efficacy of 5% 5-FU cream for intravaginal treatment of CIN II [62]. In the study, 60 women with CIN II were randomised to two groups, treatment (*n* = 31) and observation (*n* = 29). There was no placebo group. The group undergoing treatment was told to apply 2 g Efudex® cream at night to the cervix using a vaginal applicator and to place a tampon to maintain cream at the cervix. Efudex® cream is a treatment for multiple actinic or solar keratosis. Dosing was once every 2 weeks for a total of eight doses. The results of this study clearly showed a significant increased likelihood of CIN II regression for patients treated with topical 5-FU cream. A total of 93% of the treated group demonstrated regression compared to 56% for the observed group. After 6 months, the treated group was twice as likely to be both HPV negative and clear of neoplasia. The study also demonstrated that the dosing regimen of 5-FU cream was well tolerated. However, the clinicians caution that there was potential increased risk of sexually transmitted infections due to the treatment, and the patients had to understand the importance of effective birth control during treatment.

Imiquimod is an immunomodulator which triggers a local immune response effective against both cancer cells and viruses [63]. The drug increases the potency of natural killer cells by increasing the levels of cytokines in a local area. The drug also boosts the adaptive immune systems, increasing the action of both tumour-specific T cells and antibody producing B cells. Topical IMQ cream is used to treat basal cell carcinoma, actinic keratosis and genital warts. Off-label treatments have been investigated for intraepithelial neoplasia of the vulva and vagina [64]. Two clinical studies have been reported on the use of the cream to treat high-grade CIN (II/III). Grimm et al. investigated the off-label use of topical IMQ cream as an alternative to surgical intervention [65]. The study enrolled 59 patients into two randomised groups, placebo (*n* = 29) and treated (*n* = 30). Treatment involved vaginal suppositories of Adeps solidus containing 6.25 mg IMQ. For the first 2 weeks, patients applied one suppository per week; for weeks 3 and 4, patients applied two suppositories weekly; and for the final 12 weeks, the patients applied three suppositories weekly. The suppositories were applied prior to going to bed. The main outcome was efficacy of the treatment, which was defined as histological regression of to CIN I or less within 4 weeks of treatment cessation. The primary end point was observed for 73% of the treated group, compared to 39% of the placebo group. A total of 60% of the treated group were also HPV negative compared to only 14% of the placebo group post-treatment. Indeed, IMQ treatment also demonstrated a 60% clearance against the high-risk, aggressive HPV-16 strain compared to 6% for the placebo arm.

The second clinical study by Koeneman et al. began in January 2015 [66]. The study aimed to enrol 140 women with CIN II/III randomised to two groups, IMQ treatment (*n* = 70) and standard treatment (*n* = 70). Treatment would be over 16 weeks involving vaginal application of 5% IMQ cream (12.5 mg) three times per week. Primary end point was defined as regression to CIN I or less at 4 weeks after treatment cessation. Patients were to be monitored at regular intervals up to 24 months after treatment. However, the study had to be preliminarily stopped in May 2016 due to lagging inclusions of women who had strong preferences for either one of the two treatments. A study by the authors found that in most cases, women prefer the standard LLETZ treatment as it provides a fast and effective treatment of high-grade CIN [67]. On the other hand, women hoping to maintain the strongest chance of future pregnancy wished to pursue the non-surgical IMQ treatment. The trial has now converted to an open-label, non-randomised, controlled study to evaluate efficacy of topical IMQ treatment [68]. The clinical team aims to develop a biomarker profile to predict patients who would have the best clinical response to IMQ treatment. Recruitment began in November 2016.

### Vaginal gels

A range of other vaginal dosage forms and devices have been investigated for the localised treatment of cervical cancer. Bilensoy et al. developed thermosensitive mucoadhesive gels containing 5-FU in a complex with cyclodextrin (CD) [69]. The gels had a lower viscosity at room temperature and thus were easier to administer to the cervix through the vagina. However, upon administration and exposure to the higher temperature in the body, they increased in viscosity, providing a sustained release of the 5-FU CD complex (5-FU:CD), while their mucoadhesive properties ensured longer residence at the cervix. Furthermore, cytotoxicity studies demonstrated that 5-FU:CD, which only contained 25% 5-FU, was equally effective against HeLa human cervical carcinoma cells as the same dose of free 5-FU, indicating better therapeutic efficacy using a lower dose of 5-FU [69]. Debata et al. developed a curcumin-based vaginal cream that had the potential to eliminate a variety of HPV positive cervical cancers and had no adverse side effects when tested in mice [70]. A study demonstrated that a 1% cidofovir vaginal gel was effective against CIN III [71]. Seven of the 15 patients tested showed complete inhibition of the cervical dysplasia lesions after only three applications (every other day), while five showed a partial response, characterised by the persistence of CIN II-III lesions, one patient had a lower grade dysplasia (CIN I) and two patients had no response. The effect was specific and no tissue other than the dysplastic epithelium was affected by the treatment [71]. A phase II double-blind, placebo-controlled study of a 1% cidofovir vaginal gel in HPV positive patients demonstrated that no patients in cidofovir group experienced progression of the disease compared to 45% of patients in the placebo group [72]. These two clinical studies taken together demonstrate the benefits of using a vaginal gel, for localised drug delivery, to stop progression to cervical cancer by either eradicating or down staging precancerous CIN or stopping HPV infections progressing to CIN.

### Vaginal tablets

Baffoe et al. describe the manufacture and testing of both immediate and sustained release vaginal tablets containing 130 mg of the drug disulfiram [73]. Disulfiram has been used safely in the clinic for many years to treat alcoholism and has shown potential anti-tumour activity as it can induce apoptosis in some cell lines and reduce cell growth in certain tumours [74]. An anticancer effect has been demonstrated in prostate cancer, breast cancer, lung cancer, leukaemia and cervical adenocarcinoma [75–83]. Baffoe et al. demonstrate that disufiram has a dose-dependent inhibition of the HeLa cervical cancer cell line with an IC_50_ value of 124.3 nM, which is significantly more therapeutic than cisplatin with an IC_50_ value of 2.2 μM against the same cell line. Furthermore, they show, using a bio-relevant dissolution model, that their immediate and sustained release tablets can release disulfiram at 25,000 to 35,000 times its IC_50_ concentration [73]. A multicentre observational study in 256 patients with histologically proven CIN I demonstrated a 75.5% regression rate (the CIN was completely eradicated) to a negative histology after twice-weekly administration of bovine-colostrum containing vaginal tablets (Ginedie®) for 6 months [84]. Another study in 20 women positively diagnosed with HPV16 infection and early stage CIN demonstrated complete elimination of the HPV infection and early stage CIN in 60% of patients treated with one course of praneem, a polyherbal containing vaginal tablet, compared to just 10% in the placebo group [85]. Both of these clinical studies further demonstrate the potential of localised drug delivery in stopping HPV and early stage CIN from progressing into cervical cancer.

### Vaginal rings

Vaginal rings are torus-shaped drug delivery devices that are capable of providing controlled delivery of substances to the vagina for up to a period of 1 to 12 months where it slowly releases one or more drugs to provide either a local or systemic effect [86–89]. Vaginal rings have already seen clinical and commercial success in contraception (Nuvaring®) [88, 90, 91] and oestrogen replacement therapy (Estring® and Femring®) [86, 92]. Femring® and Estring® are both manufactured from silicone elastomer, whereas Nuvaring® is manufactured from ethylene-vinyl-acetate EVA. The clinical and commercial success of these rings makes them ideal candidates for the delivery of chemotherapeutic drugs to the cervix. The vaginal ring overcomes many of the disadvantages associated with gels, tablets and pessaries, which are often messy, interfere with intercourse and are poorly retained within the vagina. Boyd et al. describe the development of a disulfiram releasing vaginal ring that provides diffusion controlled release of DSF for 14 days at levels well in excess of its IC_50_ value for the HeLa cervical cancer cell line [93]. This ring has the potential to release disulfiram for up to 90 days if required. Keskar et al. developed a cisplatin vaginal ring that is capable of releasing cisplatin at levels well in excess of its IC_50_ for up to 90 days [43]. In vitro testing of the vaginal rings found them to be just as effective against both HPV positive and HPV negative cervical cancer, when compared to cisplatin alone [43]. This study demonstrates that local drug delivery of cisplatin to treat cervical cancer has the potential to be just as effective as systemic delivery.

### Cervical caps

The cervical cap is a barrier method of contraception that is placed over the cervix preventing sperm from entering and fertilising the egg. The vaginal sponge is also used for contraceptive and is a soft single use device which is soaked in a spermicide such as nonoxynol-9. A phase II clinical trial of β-All-trans-retinoic Acid (atRA) delivered via a cervical cap/vaginal sponge combination in 20 patients demonstrated 50% regression of CIN, with mild systemic and cervical side effects and moderate, but tolerable, vaginal side effects [41]. A randomised phase III clinical trial of atRA delivered via a cervical cap/vaginal sponge combination in 301 patients (151 patients with CIN II and 150 patients with CIN III) demonstrated a CIN II regression rate of 43% in the atRA group compared to just 27% in the placebo group. However, there was no significant difference between the two arms in the CIN III patients, demonstrating that the localised delivery of atRA is effective against moderate CIN but not severe CIN [94]. A similar result was demonstrated by Ruffin et al. who performed a dosing study of atRA delivered via a cervical cap/vaginal sponge combination in 175 women with either CIN II or CIN III. They found that low doses of atRA were no more effective than the placebo. However, participants with CIN II at baseline were more likely to be free of disease at 12 weeks than participants with CIN III at baseline [95].

Taken together, these three clinical studies demonstrate that the use of a cervical cap/sponge combination to deliver an appropriate dose of a chemotherapeutic drug directly to the cervix can cause CIN I and II to significantly regress and in some cases be completely eradicated, thus reducing the possibility of the patient developing cervical cancer. However, regression for CIN III was less significant, which may be more to do with the active ingredient rather than the method of delivery. A study using the novel cervical cap CerviPrep™, which was specifically designed for the direct application of pharmaceuticals to the cervix, demonstrated that it could deliver pharmacologically relevant concentrations of gemcitabine to the cervix of 11 out of the 16 women tested, with neither gemcitabine nor its metabolites being detected in the peripheral plasma of any subject [96]. This study demonstrates that it is possible to deliver effective levels of chemotherapeutic drugs to the cervix using localised delivery, while limiting plasma levels thus reducing systemic toxicities.

### Bioadhesive patches

A bioadhesive patch is a drug-loaded patch that is designed to adhere to the surface of biological tissue, where it provides sustained topical delivery of a drug within a defined surface area. For the localised treatment of cervical cancer, the patch would be applied to the cervix where it would release the chemotherapeutic drug. Woolfson et al. describe the development of a novel bioadhesive cervical patch that was capable of providing substantial drug release of the chemotherapeutic drug 5-FU through human cervical tissue samples for over 20 h [40]. The same group subsequently evaluated the release of 5-FU from the patch into cervical tissue and found that tissue concentrations were 100-fold the concentration required for 5-FU to be cytotoxic against HeLa cervical cancer cells [97]. These two studies demonstrate that by using a bioadhesive cervical patch, which actually adheres to the surface of the cervix, it is possible to deliver effective levels of the drug into the cervical tissue overcoming some of the issues associated with drug diffusion through vaginal mucus.

Sidhu et al. conducted a randomised double-blind controlled clinical trial on a bioadhesive patch containing the drug 5-FU [98]. The trial only enrolled patients in which the whole transformation zone was visible and who had CIN I/II; CIN III patients were excluded on ethical grounds. One hundred patients were tested with a 5-FU-loaded patch for a 24-h period. The patch was bilayer containing a vinyl backing onto which a drug-loaded film was bonded. The film layer was produced through casting from a 2% *w*/*w* Carbopol® gel. The patch was sufficiently flexible to conform to the shape of the entire cervix. The patch fulfilled the requirements for the treatment of CIN I/II without causing any architectural damage. However, the single dose of 5-FU did not provide effective treatment of the disease, even though 66% of the 5-FU was released in the first 28 min and penetrated the cervical tissue to a depth of 5.5 mm.

The author’s explanation for the lack of efficacy is due to the placebo-controlled group having a 72% spontaneous regression rate of CIN I/II. The study design required that the spontaneous regression rate was less than 60% with placebo. The spontaneous regression rate was significantly higher than previously reported in other studies (14–45%) [99–101]. Newer studies have shown regression rates of up to 58% over a 2-year period for CIN I [102]. Even the most persistent high-grade lesions CIN II and CIN III have reported rates of spontaneous regression from 6 to 50%, with rate dependent on diagnosis, and length of time since initial diagnosis [103]. Although not definitive, there is mounting evidence that spontaneous regression of CIN is patient-dependent and primarily determined by the patient’s local immune response to HPV [104–107].

A further factor postulated by the authors in the study may be related to the choice of polymeric material to make the film and that the placebo patch could in itself have aided regression. The polymer selected was Carbopol®, a resin that has an approximate pH range of 2.8 to 3.2 in solution. Normal vaginal pH is 4.5 [108]. Thus, this altered pH at the cervical epithelium may have been sufficient to cause cell death, increasing regression rate. The authors suggest that a future trial may yield better results should CIN III patients be included, and a control group who would not receive a placebo patch. The same group described the development of a bioadhesive patch containing IMQ that released significantly more drug across a model membrane than the proprietary IMQ cream over a 6-h period [109]. IMQ dosing can vary when using the proprietary cream due to difference in application methods and the amount of cream applied. Since the patches contain a defined drug loading per unit area, they have the potential of minimising the variations in dosing that is associated with the cream. Other bioadhesive formulations such as pellets are currently being investigated for the localised treatment of cervical cancer [110].

### Particulate drug delivery systems

Particulate drug delivery systems, such as nanoparticles and liposomes, have been investigated for vaginal drug delivery since they have a number of advantages over more typical drug depot-based dosage forms. These advantages include improved sustained release, controlled vaginal absorption, and improved cellular uptake and targeting [111]. Although the majority of the research on particulate drug delivery systems for vaginal drug delivery has focused on HIV prevention, there have been some examples of it being investigated for the localised treatment of cervical cancer. Yang et al. describe the development of biodegradable mucus-penetrating particles (MPP) that consist of particles coated with Pluronic F127, which are capable of diffusing rapidly through spaces in the mucus mesh, compared to uncoated particles which are immobilised in mucus through adhesive interactions [112]. Paclitaxel-loaded MPPs were shown, using a cervical cancer mouse model, to distribute throughout the vaginal mucus, coming in close contact with the tumour, whereas uncoated particles aggregated in the mucus and were kept away from the tumour. As a result, the paclitaxel-loaded MPPs were more effective at suppressing cervical tumour growth and almost doubled the median survival time of the mice [113]. These MPPs offer a new and promising platform for the localised treatment of cervical cancer and may overcome some of the issues associated with drug diffusion through vaginal mucus.

Zeng et al. developed nanoparticles consisting of a cholic acid functionalised, star-shaped block copolymer of PLGA and vitamin E TPGS for the sustained and controlled delivery of docetaxel [114]. These nanoparticles had superior in vitro and in vivo performance when compared with conventional PLGA nanoparticles and linear PLGA-b-TPGS copolymer nanoparticles, with a significantly higher cellular uptake and antitumour efficacy [114]. This may be as a result of their smaller size when compared to the conventional PLGA nanoparticles and linear PLGA-b-TPGS copolymer nanoparticles [114], demonstrating the importance of size when it comes to formulating nanoparticles for the localised treatment of cervical cancer.

Saengkrit et al. demonstrated increased cellular uptake and cytotoxicty of liposomal curcumin compared to free curcumin in both HeLa and SiHa cell lines [115]. Furthermore, by making the liposomal curcumin cationic through surface modification with various compositions of didecyldimethylammonium bromide (DDAB), cholesterol and non-ionic surfactant increased its anticancer efficiency. This study demonstrates the potential of liposomal formulations in enhancing the cellular uptake of hydrophilic drugs improving their anticancer efficiency and through surface modification of the liposome, cellular uptake and anticancer efficiency can be further enhanced. Chen et al. compared paclitaxel-loaded multi-layered liposomes with the same liposome only coated with anionic polyacrylic acid (PAA) followed by cationic chitosan [116]. They demonstrated that the coated liposomes exhibited a more sustained release of paclitaxel as well as an enhanced cytotoxicity in human cervical cancer cell lines compared to the uncoated liposomes [116]. This is further evidence, which demonstrates that the anticancer efficiency of particulate drug delivery systems can be enhanced through either modifying or coating their surface. However, significant variability has been observed, in both preclinical and clinical studies, of tumour accumulation and intra-tumoural distribution with liposomes [117–120]. Stapleton et al. demonstrated using two mouse xenograft tumour models of cervical cancer that regions with higher tumour perfusion have a greater degree of liposome accumulation [121]. These findings demonstrate that by determining tumour perfusion, it may be possible to select patients who are more likely to respond to liposome and other particulate-based therapies.

## Conclusion

Cervical cancer is the third most prevalent cancer in women globally and is especially dominant in developing countries due to a lack of screening, prevention and control programs. Treatment depends on the stage of the cancer and whether or not the woman would like the option to be able to have children after treatment. Most treatment regimens will involve surgery to remove the cancerous tissue, but the technique may vary. Some treatment regimens, particularly those used to treat more advanced stage cancers, may involve additional adjuvant chemo or radiotherapy. Vaginal drug delivery devices and formulations could be utilised to provide localised delivery of chemotherapeutic drugs to the cervix.

As of yet, none of the drug delivery systems described in this review are approved for first-line treatment of precancerous or early stage cervical cancer. Nevertheless, localised delivery of chemotherapeutic drugs directly to the cervix has the great potential to improve patients’ overall quality of life both during and after treatment as a result of direct delivery to the site of action, which results in a lower dose being required and a reduction in systemic side effects. Certainly, since there is an increasing reluctance to treat CIN I, CIN II and even CIN III surgically in young women who wish for future pregnancy, the most logical approach is to find pharmacological means to reduce the size or completely eradicate the abnormal tissue. Precision medicine is another incentive for localised delivery, as this approach will require treatment with combination therapy to knockout the multiple molecular pathways that support cancer cell growth.

Furthermore, since cervical cancer is such a significant burden in the developing world, there must be additional motivation in the research community to develop a delivery system that can underpin cryotherapy and do so in the context of single visit treatments. The most logical approach would be to learn lessons from the microbicides field and the fight against the HIV epidemic. Vaginal rings have been shown to be a cost-effective and efficacious means for controlled drug delivery in low-resource settings [122]. Can a vaginal ring or a similar device be given to patients in low-resource settings that will provide a level of treatment sufficient to increase regression rates and hence lower mortality rates in these countries?

It is not so much a device that requires significant development, but instead, it is the identification of a cocktail of well-tolerated drugs that can be combined in a single device that can be safely worn for extended periods of time and with limited clinical intervention. Disulfiram could be one such drug, given its long history in clinical use, but there are even more drugs that could be suitable for such repurposing, including aspirin, metformin and itraconazole, which are being investigated for anti-cancer properties. It is our contention that there already exists the framework for an intravaginal device for the treatment of cervical cancer in the developing world. Instead what is required is the willpower of the funding bodies and the research community to articulate and bring to fruition a better, more effective means of treatment that can run in tandem with existing efforts to increase screening of the disease, and consequently drive down the dreadful mortality rates in these countries.
